# Identification of Etiology-Specific Diarrhea Associated With Linear Growth Faltering in Bangladeshi Infants

**DOI:** 10.1093/aje/kwy106

**Published:** 2018-05-15

**Authors:** Amanda E Schnee, Rashidul Haque, Mami Taniuchi, Md Jashim Uddin, Md Masud Alam, Jie Liu, Elizabeth T Rogawski, Beth Kirkpatrick, Eric R Houpt, William A Petri, James A Platts-Mills

**Affiliations:** 1 Division of Infectious Diseases and International Health, School of Medicine, University of Virginia, Charlottesville, Virginia; 2 International Centre for Diarrhoeal Disease Research, Bangladesh, Dhaka, Bangladesh; 3 Department of Medicine and Vaccine Testing Center, College of Medicine, University of Vermont, Burlington, VT

**Keywords:** children, diarrhea, growth, qPCR, stunting

## Abstract

Childhood diarrhea in low-resource settings has been variably linked to linear growth shortfalls. However, the association between etiology-specific diarrhea and growth has not been comprehensively evaluated. We tested diarrheal stools collected from the Performance of Rotavirus and Oral Polio Vaccines in Developing Countries study from 2011 to 2013 in Dhaka, Bangladesh, by quantitative polymerase chain reaction for a broad range of enteropathogens to characterize diarrhea etiology and examine the association between etiology-specific diarrhea and linear growth and systemic inflammation. Pathogen-specific burdens of diarrhea were determined using attributable fractions. Linear regression was used to examine associations of pathogen-specific diarrhea with length-for-age *z* scores (LAZ) and serum C-reactive protein. There was no relationship between all-cause diarrhea and length at 12 months (change in 12-month LAZ per episode, −0.01, 95% confidence interval (CI): −0.06, 0.03). However, *Cryptosporidium* (change in 12-month LAZ per attributable episode, −0.23, 95% CI: −0.50, 0.03), *Campylobacter jejuni/coli* (change of −0.16, 95% CI: −0.32, −0.01), and *Shigella*/enteroinvasive *Escherichia coli* diarrhea (change of −0.12, 95% CI: −0.26, 0.03) were associated with linear growth deficits. Diarrhea attributable to *C. jejuni/coli* and *Shigella*/enteroinvasive *E. coli* were associated with elevated C-reactive protein. The association between diarrhea and linear growth appears to be pathogen-specific, reinforcing the need for pathogen-specific interventions.

Despite a reduction since 1990 in acute mortality attributable to diarrhea in children under age 5 years, diarrhea incidence has declined more slowly (1). Consequently, the morbidity associated with diarrheal disease, including long-term sequelae such as poor child growth and cognitive development, is of continued importance (2–4). Diarrhea, a syndrome caused by a broad range of viruses, bacteria, and parasites, has been associated with poor growth in some studies but not others (2, 3, 5). Identifying the etiology of a diarrheal episode is difficult; the clinical presentation is poorly differentiating, and multiple pathogens are frequently detected in a single diarrheal stool specimen, confounding etiologic attribution.

The association between etiology-specific diarrhea and child growth has not been comprehensively evaluated, and previous studies have focused on one or a few pathogens (6–11). However, *Campylobacter*, *Shigella*, enterotoxigenic *Escherichia coli*, and *Cryptosporidium* have been variably identified as agents of diarrhea associated with growth shortfalls. These studies have defined etiology-specific diarrhea as any episode of diarrhea in which the pathogen of interest is detected without adjustment for subclinical carriage of enteropathogens, and use of increasingly sensitive detection methods has revealed a striking burden of subclinical enteric infection in these settings (12). As a result, there has been increased use of methods that account for subclinical pathogen carriage to attribute diarrhea to specific pathogens (1, 13, 14). Quantitative polymerase chain reaction (qPCR) has increased the specificity of pathogen attribution because higher pathogen quantities are more strongly associated with diarrhea (15).

Environmental enteric dysfunction is a syndrome defined by subclinical inflammation and changes to small intestinal architecture and is thought to play a role in oral vaccine failure as well as cognitive delays and growth shortfalls (16). The Performance of Rotavirus and Oral Polio Vaccines in Developing Countries study (PROVIDE) is a longitudinal birth cohort study developed to understand the relationship between environmental enteric dysfunction and oral vaccine performance using a randomized trial of oral vaccines (17). We tested all available stool specimens collected from diarrheal episodes identified via household surveillance in the first year of life, using qPCR for a broad range of enteropathogens. In the present study, we estimated pathogen-specific burdens of diarrhea and the association between etiology-specific diarrhea and both linear growth and systemic inflammation in this cohort.

## METHODS

### Study design

Detailed methods of PROVIDE have been reported elsewhere (17, 18). Briefly, the study was conducted at a single site in the Mirpur area of Dhaka, Bangladesh. Children were enrolled within 7 days of birth and followed until age 2 years. A survey was performed to collect data about the child’s environment as well as sociodemographic data. Children were randomized by 2 × 2 factorial design to receive rotavirus vaccine at 10 and 17 weeks of age or to have injectable polio vaccine in place of the standard fourth dose of oral polio vaccine at 39 weeks of age. Enrolled families were visited biweekly to identify the duration of exclusive breastfeeding, antibiotic use, and diarrheal episodes, from which stool samples were collected. Diarrhea was defined as 3 or more abnormally loose stools within 24 hours, and episodes were separated by a minimum of 72 diarrhea-free hours. Anthropometry measures were collected at enrollment and 12, 24, 40, 52, and 104 weeks of age. Serum was collected for high-sensitivity C-reactive protein (CRP) testing at 6, 18, 40, and 53 weeks of age. Written consent was obtained from the parent or guardian of each enrolled child. The study was approved by the ethical review boards of the International Centre for Diarrhoeal Disease Research, Bangladesh, the University of Vermont, and the University of Virginia. The study was registered at ClinicalTrials.gov (NCT01375647).

### Laboratory testing

CRP testing was conducted at International Centre for Diarrhoeal Disease Research, Bangladesh, using the CRP ELISA Kit (Immundiagnostik AG, Bensheim, Germany), following the product’s standard operating procedure. Stool total nucleic acid was extracted with the QIAamp Fast DNA stool mini kit (Qiagen, Hilden, Germany), using a modified protocol that included bead beating (9). As extrinsic controls, phocine herpesvirus (PhHV) and bacteriophage MS2 were spiked to the lysis buffers to monitor extraction and amplification. Extraction banks and no-template controls were included to monitor for contamination. The qPCR testing was performed via a custom TaqMan Array Card (Thermo Fisher Scientific, Carlsblad, California) (19). Enteropathogen targets on the card included all of the pathogens analyzed by qPCR in the Global Enteric Multicenter Study (GEMS), and diarrhea episodes included in the analysis were required to have complete, valid data for all pathogens that were found to be etiologic in the GEMS (i.e., adenovirus 40/41, *Aeromonas*, astrovirus, *Campylobacter jejuni/coli*, *Cryptosporidium*, *Cyclospora*, *Entamoeba histolytica*, *Helicobacter pylori*, heat-labile toxin-producing enterotoxigenic *E. coli*, norovirus genogroup II, rotavirus, *Salmonella*, *Shigella*/enteroinvasive *E. coli*, heat-stable toxin-producing enterotoxigenic *E. coli*, Shiga toxin-producing enterotoxigenic *E. coli*, typical enteropathogenic *E. coli*, and *Vibrio cholerae* (15).

### Statistical analysis

To calculate pathogen-specific burdens of diarrhea in the cohort, we calculated an adjusted attributable fraction (AF) of diarrhea for each pathogen. Specifically, we used a model developed for the Bangladesh site in the GEMS to derive quantity-specific odds ratios for the association between each pathogen and diarrhea (15). Using qPCR data from 877 cases of moderate-to-severe acute diarrhea and age-, sex-, and village-matched controls in the GEMS, we fitted a multivariable conditional logistic regression model to describe the association between pathogen quantity (using linear and quadratic terms) and diarrhea while adjusting for the presence of other pathogens. We then calculated AFs by summing the pathogen-attributable fraction for each episode (AFe) across each of *j* cases in the PROVIDE study (i.e., $\sum_{1}^{j}\left(1/j\times AFe_{i}\right)$, where $AFe_{i}=1\;–\;1/OR_{i}$, and *OR*_*i*_ is the quantity-specific odds ratio derived from the regression model). To estimate the variance for the model-based attribution, the odds ratios were estimated 10,000 times using random perturbations of the model coefficients in accordance with their sampling variance-covariance. We derived 95% confidence intervals from the 2.5th and 97.5th quantiles of the AF distribution, and the point estimate of the AF was calculated using the original model coefficients. The point estimate was also used to calculate AFe for each individual episode of diarrhea.

To estimate the association between all-cause diarrhea and linear growth, we fitted a multivariable linear regression model with an outcome of length-for-age *z* score (LAZ) at 12 months. In addition to either total number of diarrheal episodes or days of diarrhea, the covariates were LAZ at enrollment; the child’s sex; maternal age, height, and education; household monthly income; household crowding (defined as more than 5 individuals living in the home); rotavirus and polio vaccination arm; presence of a flush toilet; routine treatment of drinking water; presence of a cement floor in the home; presence of a kitchen within the home; duration of exclusive breastfeeding; and antibiotic treatment of each episode. Finally, because etiology-specific diarrhea is seasonal, and the association between enteric pathogens and child growth might depend on the age at exposure, seasonality was modeled via a second-order Fourier series by including the periodic terms *sin*(2*m*π/12), *cos*(2*m*π/12), *sin*(4*m*π/12), and *cos*(4*m*π/12), where *m* is the child’s month of birth. To estimate the association between diarrhea etiology and linear growth, the same model was used but with the addition of the sum AFe across all tested episodes of diarrhea for each child, first for pathogen type (bacterial, viral, or protozoal) and then for individual enteropathogens, with pathogens selected for inclusion based on attributable burden as well as prior evidence of an association with growth. The total number of diarrheal episodes was retained in this model. As a secondary analysis, we scaled the coefficients to reflect the difference in low (10th percentile) and high (90th percentile) burdens of pathogen-attributable diarrhea. These models were repeated with the outcome of 24-month LAZ instead of 12-month LAZ. To assess the relative association between etiology-specific diarrhea and growth in the first and second 6 months of life, the AFe sums were recalculated for both time periods, which were then included in an otherwise-identical model.

To assess whether prior linear growth deficits were associated with subsequent etiology-specific diarrhea, which would suggest reverse causality, we used generalized estimating equations to fit a logistic regression model with robust variance to account for the nonindependence of serial stool testing for each child. The outcome was any pathogen-attributable diarrhea in the window between length measurements, and covariates included the LAZ at the beginning of the window, any pathogen-attributable diarrhea in the prior window, and all previously described subject-level covariates used in the growth models. To estimate risk ratios for etiology-specific diarrhea, we used Poisson regression as an approximation of log-binomial regression because the log-binomial models did not converge (20).

Finally, to determine whether etiology-specific diarrhea was associated with systemic inflammation as measured by serum CRP, we used generalized estimating equations to fit a linear regression model with robust variance to account for nonindependence of serial CRP measurements for each child. The predictors were all previously described subject-level covariates used in the growth models, as well as either the sum AFe for each pathogen used in the growth analysis during the interval prior to each CRP measurement or the sum AFe restricted to a 4-week interval prior to each CRP measurement. To assess for possible variations in CRP within brief periods around diarrheal episodes, we looked at exposure both in the interval between each CRP measurement and within the 4 weeks immediately prior to each measurement. CRP was converted to a log scale to fit the model and then converted back to a linear scale for reporting the coefficients. Finally, we added the log mean CRP for each child to the linear growth model to estimate the relationship between inflammation and linear growth attainment. All statistical analysis was performed using R, version 3.3.1 (R Foundation for Statistical Computing, Vienna, Austria).

## RESULTS

### Diarrhea surveillance, stool collection and testing, and follow-up

Between May 22, 2011, and November 6, 2012, 700 children were enrolled. Among all enrolled children, there were 2,559 episodes of diarrhea identified by active surveillance up to 1 year of age. A stool sample was successfully obtained from 1,993 of 2,559 (77.9%) episodes. Episodes with a sample obtained were of longer duration than uncaptured episodes (median of 4 days, interquartile range, 3–6, vs. median of 1 day, interquartile range, 1–2) (Wilcoxon rank test *P* < 0.001). Of these, 1,984 of 1,993 (99.5%) had sufficient stool available for nucleic acid extraction and qPCR testing, and valid qPCR results were obtained for 1,791 of 1,984 (90.3%). For this analysis, we excluded children who did not have complete anthropometry at 52 weeks due to withdrawal from the study (*n* = 49), loss to follow-up (39 children), death (5 children), or anthropometry data not having been obtained (6 children) (Web Figures 1 and 2, available at https://academic.oup.com/aje). Excluded children were born to younger mothers with a lower household income (Table 1). For the 603 children with complete follow-up included in our analysis, 521 of 603 (86.4%) contributed at least 1 episode of diarrhea to a total of 1,741 episodes with valid qPCR results. Repeat anthropometry was available at 24 months of age for 575 of 603 (95.4%) children. Children’s lengths were generally below the World Health Organization reference standards at enrollment and decreased over time (LAZ, mean = −0.91 (standard deviation, 0.88) at enrollment; −1.46 (standard deviation, 1.00) at 12 months of age; and −1.60 (standard deviation, 1.02) at 24 months of age).

**Table 1.** Characteristics of the Study Population, Performance of Rotavirus and Oral Polio Vaccines in Developing Countries Study, Bangladesh, 2011–2013

Characteristic	Included in Analysis (*n* = 603)				Excluded From Analysis (*n* = 97)				*P* Value^a^
	No.	%	Mean (SD)	Median (IQR)	No.	%	Mean (SD)	Median (IQR)	
Child’s characteristics									
Male sex	315	52.2			53	54.6			0.741
Vaccine									
Received rotavirus vaccine	305	50.5			45	46.4			0.512
Received IPV	299	49.6			51	52.6			0.662
Enrollment anthropometry									
Weight, kg			2.8 (0.4)				2.8 (0.4)		0.936
Height, cm			48.7 (1.7)				48.8 (1.9)		0.866
Age, days				5 (4–6)				4 (3–6)	0.010
Maternal characteristics									
No formal education	169	28.0			33	34.0			0.276
Anthropometry									
Age at first pregnancy, years				18 (17–20)				18 (17–20)	0.403
Age at child enrollment, years				24 (21–28)				22 (20–26)	0.005
Weight, kg			49.6 (9.5)				–^b^		
Height, cm			150.5 (5.5)				–^b^		
Family characteristics									
Household monthly income, per 1,000 taka				10 (7–15)				8.5 (6–12)	0.002
More than 6 individuals in dwelling	208	34.5			30	30.9			0.567
One or more children <5 years of age in dwelling	163	27.0			25	25.8			0.868
Food deficit as assessed by family member	202	33.5			37	38.1			0.435
Dwelling characteristics									
Concrete floor	556	92.2			82	84.5			0.023
State supplied potable water source	585	97.0			93	95.9			0.777
Flush toilet	576	95.5			92	94.8			0.973
Cooking space within dwelling	210	34.8			36	37.1			0.746
Routine treatment of drinking water (boiling or chlorine)	369	60.6			49	77.8			0.471

Abbreviations: IPV, injectable polio virus vaccine; IQR, interquartile range; SD, standard deviation.

^a^ χ^2^ test for dichotomous variables; Wilcoxon rank test for continuous variables.

^b^ Not available because maternal anthropometry was collected after children were excluded.

### Pathogen detection and diarrhea etiology

**Figure 1.**
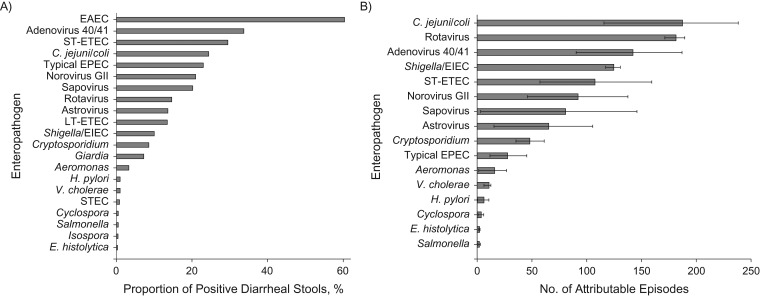
Pathogen detection and pathogen-specific etiology and burden of diarrhea in the Performance of Rotavirus and Oral Polio Vaccines in Developing Countries study, Bangladesh, 2011–2013. A) The proportion of diarrheal episodes in which each pathogen was detected by quantitative polymerase chain reaction. B) The attributable incidence. Bars represent 95% confidence intervals. *C. jejuni*, *Campylobacter jejuni*; EAEC, enteroaggregative *Escherichia coli*; *E. histolytica*, *Entamoeba histolytica*; EIEC, enteroinvasive *E. coli*; EPEC, enteropathogenic *E. coli*; GII, genogroup II; *H. pylori*, *Helicobacter pylori*; LT-ETEC, heat-labile toxin-producing enterotoxigenic *E. coli*; STEC, Shiga toxin-producing enterotoxigenic *E. coli*; ST-ETEC, heat-stable toxin-producing enterotoxigenic *E. coli*; *V. cholerae*, *Vibrio cholerae*.

**Figure 2.**
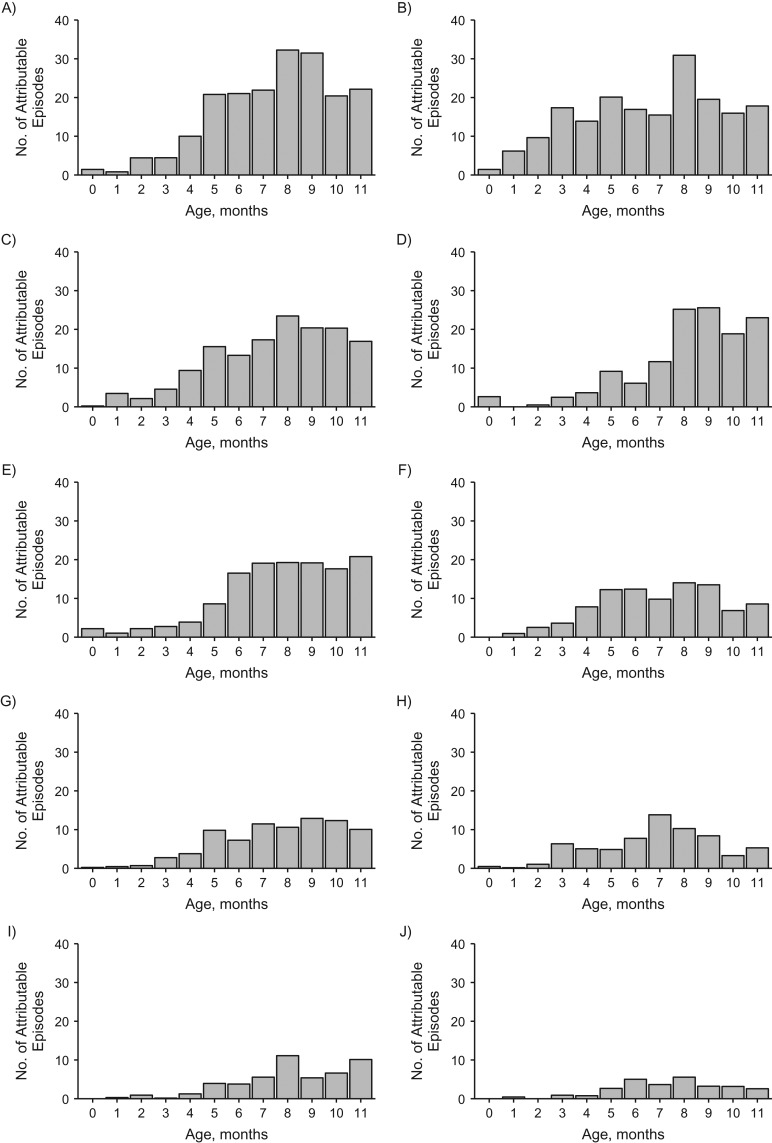
Prevalence of pathogen-attributable episodes of diarrhea according to month of age in the Performance of Rotavirus and Oral Polio Vaccines in Developing Countries study, Bangladesh, 2011–2013. Estimates are shown for *Campylobacter jejuni/coli* (A), rotavirus (B), adenovirus 40/41 (C), *Shigella*/enteroinvasive *Escherichia coli* (D), heat-stable toxin-producing enterotoxigenic *E. coli* (E), norovirus genogroup II (F), sapovirus (G), astrovirus (H), *Cryptosporidium* (I), and enteropathogenic *E. coli* (J).

A broad range of enteropathogens was detected, including 12 pathogens each detected in more than 5% of diarrheal stools (Figure 1A). Using the pathogen-specific associations with diarrhea estimated in the GEMS to determine attribution based on pathogen quantity, *C. jejuni/coli* was associated with the highest attributable incidence (187.4 attributable episodes, 95% confidence interval: 115.7, 238.3), followed by rotavirus (181.5 attributable episodes, 95% CI: 171.3, 189.5), adenovirus 40/41 (142.3 attributable episodes, 95% CI: 90.3, 187.0), *Shigella*/enteroinvasive *E. coli* (124.7 attributable episodes, 95% CI: 117.3, 130.7), and heat-stable toxin-producing enterotoxigenic *E. coli* (107.5 attributable episodes, 95% CI: 57.3, 159.4) (Figure 1B). Nineteen of 255 rotavirus detections (7.5%) were within 14 days of rotavirus vaccine administration. Diarrhea attributable to viruses (562.0 attributable episodes) and bacteria (482.6 attributable episodes) predominated, while protozoal etiologies of diarrhea were relatively uncommon (54.2 episodes). In total, 63.1% of all-cause diarrhea was pathogen-attributable. Generally, pathogen-attributable diarrhea across a broad range of etiologies peaked during 6–11 months of age (Figure 2).

### Diarrhea and linear growth

**Figure 3.**
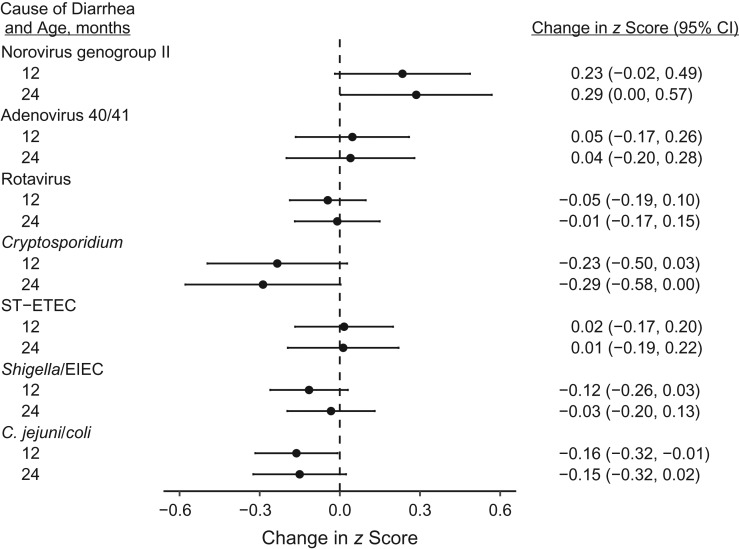
Association between etiology-specific diarrhea and linear growth attainment at 12 and 24 months in the Performance of Rotavirus and Oral Polio Vaccines in Developing Countries study, Bangladesh, 2011–2013. The effect size shown is the change in length-for-age *z* score per attributable episode. CI, confidence interval; EIEC, enteroinvasive *Escherichia coli*; ST-ETEC, heat-stable toxin-producing enterotoxigenic *E. coli*.

Neither the number of diarrheal episodes (change in 12-month LAZ per additional episode of diarrhea, −0.01, 95% CI: −0.06, 0.03) nor the number of days of diarrhea (change in 12-month LAZ per additional week of diarrhea, −0.02, 95% CI: −0.07, 0.03) were associated with LAZ at 12 months. However, diarrhea attributable to bacteria (change in 12-month LAZ per additional attributable episode, −0.09, 95% CI: −0.16, −0.01) and protozoa (change in 12-month LAZ per additional attributable episode, −0.24, 95% CI: −0.49, 0.01) was more strongly associated with linear growth deficits than was diarrhea attributable to viruses (change in 12-month LAZ per additional attributable episode, −0.01, 95% CI: −0.11, 0.08). Evaluating pathogen-specific diarrhea, the association with linear growth deficits was strongest for *Cryptosporidium*-attributable diarrhea (change in 12-month LAZ per additional attributable episode, −0.23, 95% CI: −0.50, 0.03), *Campylobacter*-attributable diarrhea (change in 12-month LAZ per additional attributable episode, −0.16, 95% CI: −0.32, −0.01), and *Shigella*-attributable diarrhea (change in 12-month LAZ per additional attributable episode, −0.12, 95% CI: −0.26, 0.03) (Figure 3). Because *Campylobacter* and *Shigella* were more common than *Cryptosporidium*, they were associated with a larger cumulative association with growth attainment (Web Figure 3). Diarrhea attributable to norovirus genogroup II was associated with higher length attainment (change in 12-month LAZ per additional attributable episode, 0.23, 95% CI: −0.02, 0.49). The association between *Cryptosporidium* and *Campylobacter* diarrhea (in the first year of life) and length persisted at 24 months of age (Figure 3). The association between *Campylobacter*-attributable diarrhea (−0.51, 95% CI: −0.92, −0.10) and *Cryptosporidium*-attributable diarrhea (−0.72, 95% CI: −1.83, 0.38) and growth was particularly strong for episodes occurring in the first 6 months of life; however, *Cryptosporidium*-attributable diarrhea was rare during this time period (3.5 total attributable episodes). Removal of the GEMS model–based attribution did not substantially change the identified associations (Web Figure 4). There was no evidence that chronic malnutrition was associated with subsequent detection of specific enteropathogens, which would suggest reverse causality. However, a lower length was most strongly associated with subsequent *Cryptosporidium*-attributable diarrhea (per 1 unit change in baseline LAZ, risk ratio = 1.14, 95% confidence interval: 0.91, 1.44).

### Pathogen-specific diarrhea, inflammation, and growth

**Figure 4.**
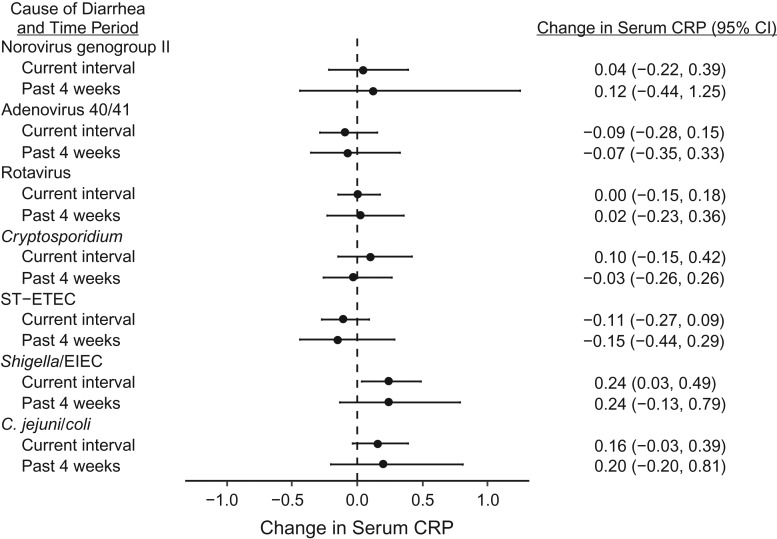
Association between etiology-specific diarrhea and serum C-reactive protein (CRP) in children at 6, 18, 40, and 53 weeks of age in the Performance of Rotavirus and Oral Polio Vaccines in Developing Countries study, Bangladesh, 2011–2013. Both the association between pathogen-attributable diarrhea during the entire interval as well as during the 4 weeks before each measurement is shown. CI, confidence interval; EIEC, enteroinvasive *Escherichia coli*; ST-ETEC, heat-stable toxin-producing enterotoxigenic *E. coli*.

Because increased systemic inflammation is a putative pathway between enteric infections and poor linear growth, we evaluated the association between pathogen-specific diarrhea and 4 CRP measurements during the first year of life. Diarrhea attributable to *Campylobacter* (increase in CRP per attributable episode, 0.16, 95% CI: −0.03, 0.39) and *Shigella*/enteroinvasive *E. coli* (increase in CRP per attributable episode, 0.24, 95% CI: 0.03, 0.49) was associated with increased systemic inflammation in comparison with other pathogens (Figure 4). Although limiting exposures to within the prior 4 weeks decreased the precision of all estimates, the magnitude of the association between pathogens and CRP increased for *Campylobacter* and *Shigella*/enteroinvasive *E. coli* and decreased for all other pathogens. There was a trend towards an association between increased systemic inflammation and linear growth faltering (change in 12-month LAZ per log increase in mean CRP, −0.061, 95% CI: −0.147, 0.024).

## DISCUSSION

In this single-site, birth cohort study in Dhaka, Bangladesh, the burden of all-cause diarrhea in the first year of life was not associated with length attainment. Although the strength of association has been variable, prior research, mostly performed several decades ago, has found that diarrhea, particularly of longer duration, was associated with poor linear growth early in life (2, 3, 21). A recent multisite birth cohort study similarly did not demonstrate this association, and it is possible that the same changes in the etiology and management of diarrhea that have reduced mortality in these settings have also attenuated the association between diarrhea and growth (1, 5). It is in this context, with no clear relationship between all-cause diarrhea and growth, that we sought to describe etiology-specific associations. To our knowledge, this is the first study to comprehensively analyze the etiology of diarrhea using molecular diagnostics and estimate the association between pathogen-attributable diarrhea and linear growth. The application of the attributable-fraction metric attempts to delineate between etiologic detections and subclinical carriage. For example, enteroaggregative *E. coli* and *Giardia* were frequently detected (Figure 1A) but not attributed as etiologic (Figure 1B). This approach is particularly important in settings with a high prevalence of subclinical enteropathogen infections.

Using this approach, we found the association between diarrhea and linear growth to be etiology-dependent. While the variance in our estimates is high, which reflects the relatively low number of individual observations available, it is unlikely that a larger, single-site cohort with the same intensity of surveillance and testing will be performed. For several reasons, we are inclined to believe that these findings are not spurious. First, our findings have significant biological plausibility. Diarrhea attributable to the 2 primary invasive bacterial agents of diarrhea, *C. jejuni/coli* and *Shigella*, as well as the protozoal small intestinal pathogen *Cryptosporidium*, was preferentially associated with linear growth shortfalls. These findings are consistent with prior single-pathogen studies that suggest that these are likely drivers of diarrhea-associated growth shortfalls (6–8, 10, 11, 22). Further, the association magnitudes identified are similar to those reported in previous studies. For example, incident *Shigella* diarrhea was associated with a growth deficit of approximately 0.15 cm per year per episode in Bangladesh in children 0–5 years of age and 0.1 cm per year in Peru in children 0–6 years of age (6, 10). Our estimate for *Shigella* of 0.12 change in LAZ at 12 months of age is equivalent to a larger association of 0.28 cm per year per attributable episode, in a study limited to infants, in whom the magnitude of the association would be expected to be greater. The finding that norovirus-attributable diarrhea burden was associated with improved length attainment was unexpected, but this might reveal that treatment for diarrhea, including antibiotic therapy, might be growth-promoting, thus suggesting that the estimated associations for other pathogens might have been attenuated.

These findings have implications for the management of childhood diarrhea, which currently suggest that antibiotic therapy be limited to dysentery, with the logic that most clinically significant shigellosis should be marked by observable blood in stool (23). However, there is evidence that a minority of *Shigella*- and *Campylobacter-*attributable diarrhea presenting for care is dysenteric, and there is no evidence that this subset is of unique clinical importance (24). *Cryptosporidium* illness can be prolonged in duration, but it is not readily identifiable based on clinical characteristics. This would suggest that point-of-care diagnostics are needed to augment current syndromic management in low-resource settings. Meanwhile, there is mounting evidence that a high burden of enteric infections, with or without associated diarrhea, is associated with growth shortfalls, which raises the question as to whether treatment targeting symptomatic infections would be insufficient (5). However, it is still possible that episodes of diarrhea are sentinel events for early enteric infections, and targeting treatment to these episodes will be of significant benefit.

The mechanisms by which these enteropathogens are associated with growth shortfalls are only partially understood. *Campylobacter* and *Shigella* infection have been associated with intestinal inflammation and permeability, hallmarks of the syndrome of environmental enteric dysfunction, and we provide evidence that they may also be associated with systemic inflammation (13, 25). There is some evidence that inflammation is an important pathway in the relationship between environmental enteric dysfunction and child growth (26). While *Cryptosporidium* infection has also been associated with intestinal inflammation, we did not identify a relationship with systemic inflammation, which has been more directly linked to linear growth shortfalls (26–29). It remains a possibility that *Cryptosporidium* is a marker, rather than a cause, of chronic malnutrition; however, we did not find evidence to support this. Further research is needed to elucidate these pathways.

Our study has several limitations. First, as a single-site study, the findings may not be generalizable. Second, because the attributable-fraction methodology discounts subclinical enteropathogen carriage, we may underestimate the relationship between these enteric infections and growth. Additionally, these models were derived from a different cohort of children, though also from Bangladesh. However, because nondiarrheal stools were not obtained, and because we specifically wanted to interrogate the relationship between diarrhea etiology and growth, we would contend that the more conservative attribution of etiology by this approach better isolates clinically apparent enteric infections. Stool collection was biased towards episodes with a longer duration, thus it is possible that we are preferentially underdetecting etiologies associated with a shorter duration. However, we suspect that the uncollected episodes, with a median duration of only 1 day, represent primarily noninfectious diarrhea and thus would not appreciably contribute to attributable pathogen burdens. Our diagnostic target for *Campylobacter* was specific to *C. jejuni* and *C. coli.* Additional species of *Campylobacter* may be associated with a significant burden of human disease and further research using a diagnostic approach that can more broadly identify *Campylobacter* species is needed (30).

In summary, our study supports the hypothesis that the association between diarrhea and poor linear growth is etiology-specific, with the burden of evidence suggesting that *Campylobacter*, *Shigella*, and *Cryptosporidium* are of particular interest. The relative importance of clinical and subclinical infections with these pathogens remains unclear, as does the benefit of antibiotic therapy. Studies of interventions, including reduced exposure, vaccination, and more precise diagnosis and management of diarrhea, are urgently required.

## Supplementary Material

Web Material

## Abbreviations

AF: attributable fraction
AFe: attributable fraction for each episode
CRP: serum C-reactive protein
GEMS: Global Enteric Multicenter Study
LAZ: length-for-age *z* score
PROVIDE: Performance of Rotavirus and Oral Polio Vaccines in Developing Countries study
qPCR: quantitative polymerase chain reaction
