# Supplementary material for: Identification of Etiology-Specific Diarrhea Associated With Linear Growth Faltering in Bangladeshi Infants
Source: Am J Epidemiol. 2018 May 15;187(10):2210–8. doi: 10.1093/aje/kwy106 (PMC6166216; doi:10.1093/aje/kwy106)

Web Material

Identification of Etiology-Specific Diarrhea Associated With Linear Growth Faltering in Bangladeshi Infants

Amanda E. Schnee, Rashidul Haque, Mami Taniuchi, Md. Jashim Uddin, Md. Masud Alam, Jie Liu, Elizabeth T. Rogawski, Beth Kirkpatrick, Eric R. Houpt, William A. Petri Jr., and James A. Platts-Mills

Web Figure 1. Child enrollment and follow-up in PROVIDE.

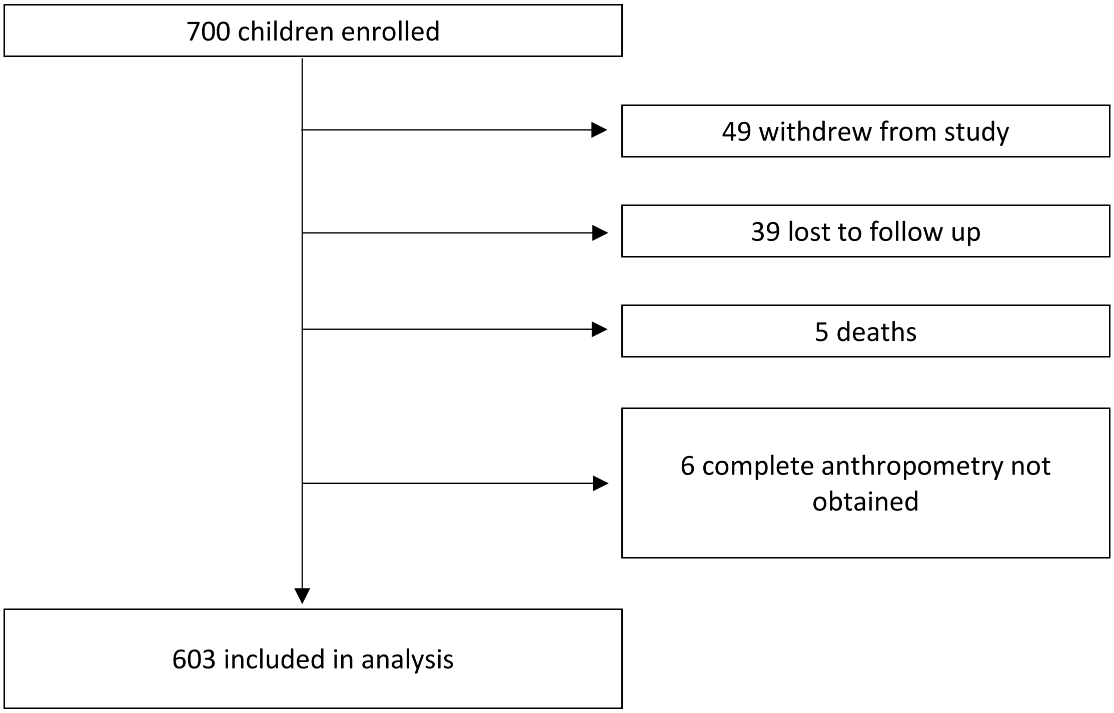

Web Figure 2. Diarrhea surveillance, sample collection, testing, and validity.

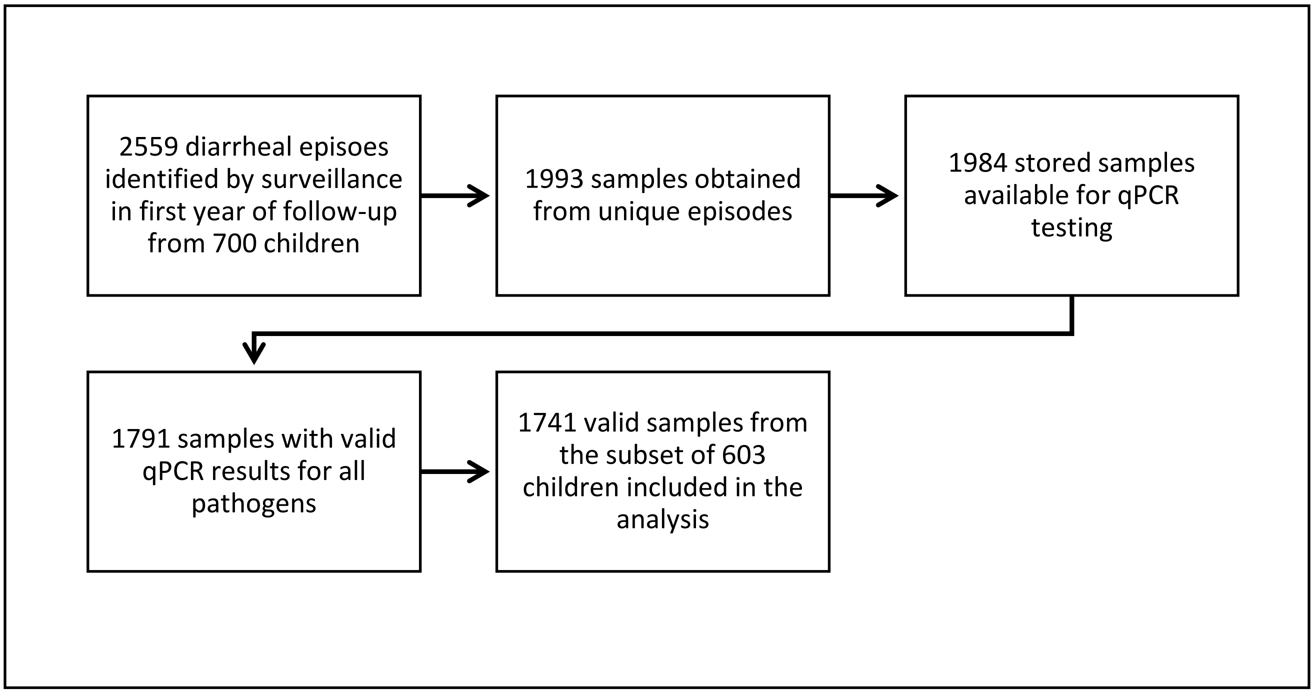

Web Figure 3. Association between a high vs low burden of etiology-specific diarrhea and linear growth attainment at 12 and 24 months.

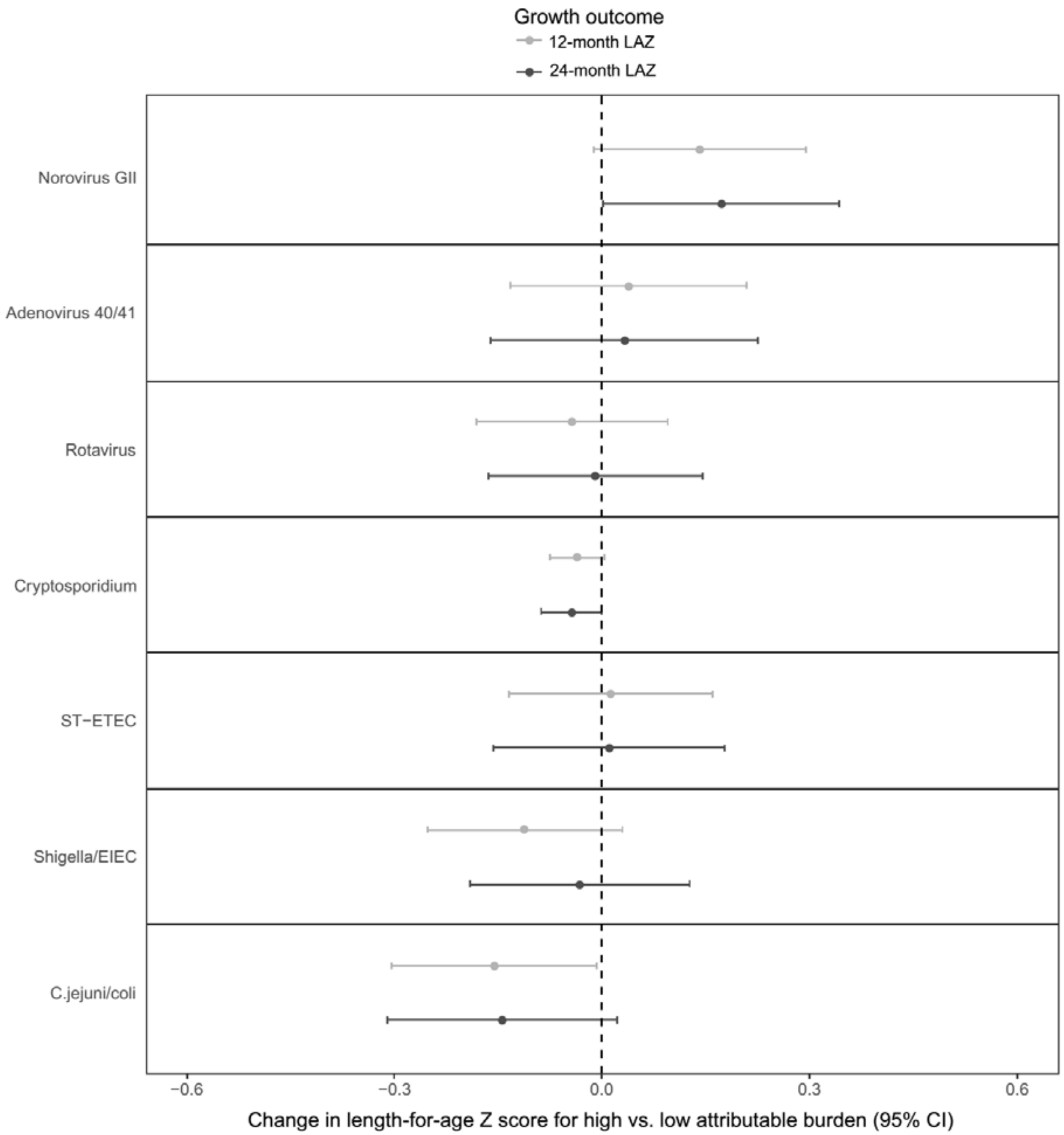

Web Figure 4. Association between diarrhea episodes with pathogen detection at any quantity and linear growth attainment at 12 and 24 months.

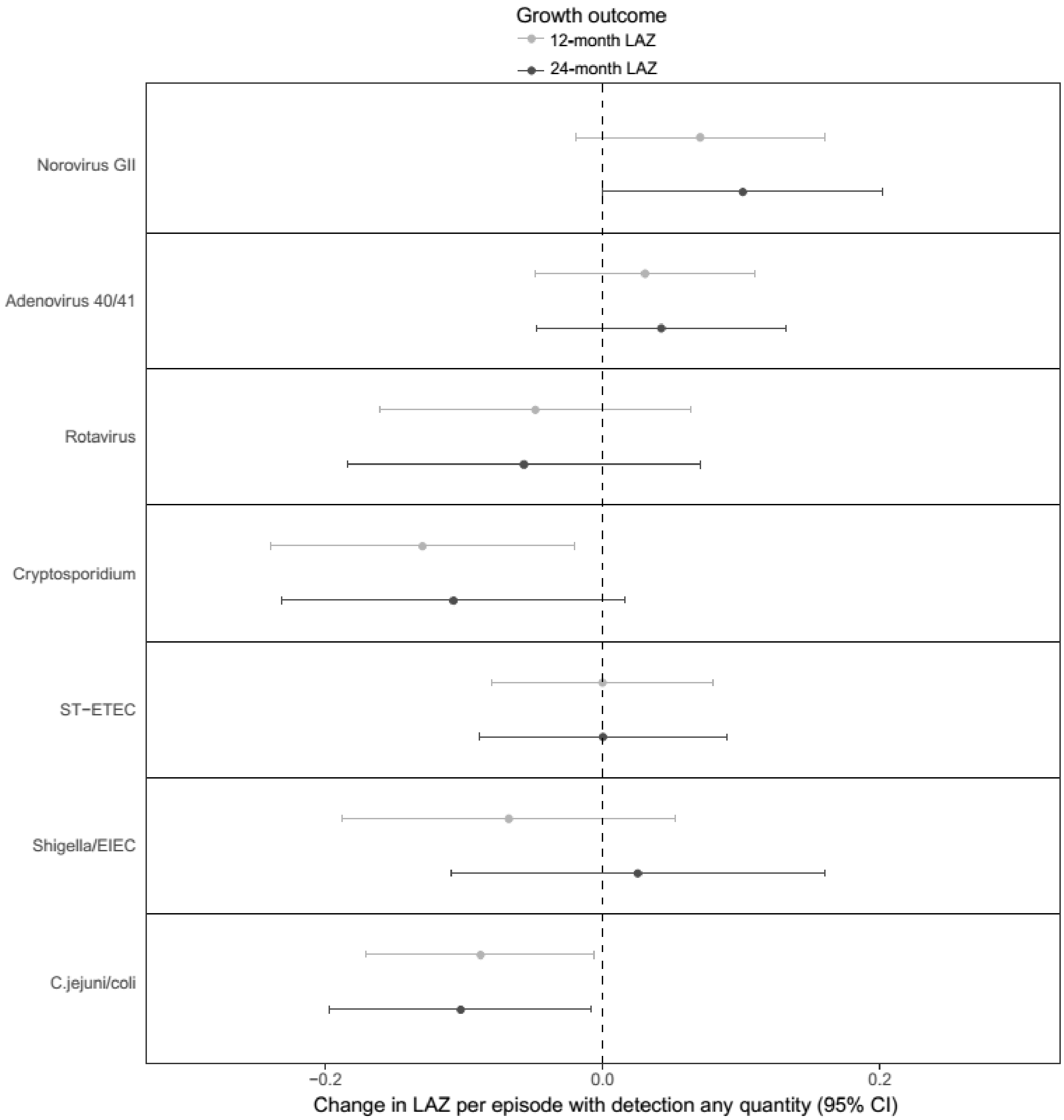

Supplement: Web Material [file kwy106_platts-mills_web_material_final.pdf]
